# How Does the Labor Protection Law Affect Sustainable Economic Growth: An Empirical Analysis Based on Psychological Contract Perspective

**DOI:** 10.3389/fpsyg.2022.928282

**Published:** 2022-08-01

**Authors:** Yina Liao

**Affiliations:** Humanities and Law School, Chengdu University of Technology, Chengdu, China

**Keywords:** Labor Contract Law, psychological contract, sustainable economic growth, enterprise investment, emerging markets

## Abstract

This article studied the influence of Labor Contract Law and employee psychological contract on enterprise investment and sustainable economic growth. The results indicate that the Labor Protection Law has no significant influence on the investment of state-owned enterprises. In the early stage of the implementation of Labor Protection Law, the Labor protection Law will observably reduce the investment level of private enterprises, and this effect is more obvious in labor-intensive industries and small and medium-sized enterprises. However, in the later stage of the implementation of Labor Protection Law, the impact of Labor protection Law on the investment of private listed companies is weak. The results indicate that the Labor protection Law increases the illegal costs of private enterprises, reduces the flexibility of employment, and ultimately reduces the investment level of enterprises. Moreover, our article examines the impact of the Labor protection Law on regional economic growth, and finds that the Labor protection Law will significantly reduce the regional GDP growth rate in China, and this effect is mainly reflected in the regions where private enterprises provide more jobs and the proportion of private economy is high. This article takes China’s emerging markets as the background, on the one hand, expands the relevant research on labor market friction from the viewpoint of enterprise investment and economic growth, on the other hand, provides new evidence for state-owned enterprises to fulfill social goals.

## Introduction

Worldwide, China’s GDP growth rate ranks first in the world during the same period, and it is known as the “growth miracle” (Xu, 2011). However, with the rapid economic growth, problems such as the contradiction between labor and enterprise also continue to appear, affecting social harmony and stability. To better protect the rights of workers and promote the construction of a harmonious socialist society, the Labor Protection Law (LPL) came into effect in 2008.

The Labor Contract Law has aroused discussions since its enactment, for example, what impact it will have on the labor cost of enterprises and whether it will affect the development of enterprises and thus China’s economic growth. Studies have shown that the Labor Contract Law with the purpose of strengthening employee protection increases the employee cost of enterprises and reduces the flexibility of employees, thus affecting the employee level, labor cost stickiness and business flexibility. These studies enrich the economic consequences of LPL from the viewpoint of enterprise labor cost and employment. However, there is no relevant evidence that whether the Labor Contract Law, which aims at promoting social harmony, affects enterprise investment at the micro level and then affects economic growth at the macro level. The importance of these issues lies in the fact that economic growth (efficiency) and social harmony (fair) are not always balanced in a country’s development process (Miller, 1959), thus the study on the impact of work Contract Law on business and financial development has significant importance.

This article analyzes whether the Labor Contract Law will reduce the investment level of enterprises and hinder China’s economic growth at the macro level. Theoretically, Labor Protection Law has two kinds of effects on enterprise investment: On the one hand, according to the view of illegal cost, Labor Protection Law has increased the illegal cost of enterprise employment (Belot et al., 2007), thus reducing the investment level of enterprises; According to the flexible employment view, Labor Contract Law weakens the right of enterprises to flexibly adjust project human resources according to the actual operation of projects, and reduces the allocation efficiency of project human resources (Rogerson, 1993). On the other hand, in the face of the impact of LPL, enterprises may take the initiative to deal with it: The view of employee efficiency holds that employees are encouraged to invest more proprietary knowledge by improving corporate culture (Fairhurst and Serfling, 2015) to increase the level of enterprise investment; The factor substitution view holds that to mitigate the influence of Labor Protection Law on enterprise labor costs, enterprises may substitute factors, that is, replace human capital with more advanced equipment, and ultimately enhance the investment level of enterprises.

(Connolly et al., 1986) showed that the formation and establishment of labor unions reduced the R&D return rate and scientific research investment of enterprises. Collective bargaining behavior organized by trade unions will increase the capital cost of enterprises, reduce the operating flexibility of enterprises (Chen et al., 2011), increase the debt ratio (Matsa, 2010), and increase the bankruptcy risk of enterprises. However, due to the relationship between the formation and influence of labor unions and the characteristics of enterprises themselves, foreign literature researches mainly focus on the influence of labor protection laws and system design on enterprise operation and other activities. Theoretically, labor protection has two kinds of effects on the employment of enterprises: inhibiting and promoting. On the one hand, labor protection improves the illegal labor costs of enterprises (Bronzini and Piselli, 2009) and reduces the efficiency of project human resource allocation. Weakened the ability of enterprises to flexibly adjust project human resources according to the actual economic development (Doms et al., 1997). However, according to the view of employee efficiency, enterprises can provide more professional training to employees and strengthen the investment and education of professional knowledge (Foster and Rosenzweig, 1996), because employees are more likely to obtain expected returns through their own efforts in daily work. Moreover, enterprises can no longer arbitrarily dismiss employees or carry out unfair distribution agreements, thus promoting employees’ enthusiasm for serious work (Acemoglu, 2002). The implementation of labor protection not only affects the employment of enterprises, but also affects the operation, investment and other activities of enterprises. The starting point of government intervention in the labor market is to safeguard workers’ rights and give consideration to fairness, but sometimes it does not achieve the desired effect. Studies have found that strict labor protection increases unemployment rate (Benhabib and Spiegel, 1994) and decreases enterprise productivity (Autor et al., 1998). Based on the influence of Wrongful Discharge Law in the American market, (Bird and Knopf, 2005; Serfling, 2016) found that the increase of labor costs brought by labor protection reduced the profit margin and business flexibility of enterprises. (Besley and Burgess, 2004) conducted a study based on the current situation of India, and found that labor protection inhibited the investment level of enterprises and ultimately reduced the total economic output. In the production process, enterprises need to readjust human resources according to different project environments, but labor protection hinders the ability of adjustment, which makes the option value of projects decline and ultimately reduces the investment level of enterprises (Samaniego, 2006). However, if the work efficiency of employees is improved, the long-term value of the project will be increased and the investment level of the enterprise will be improved (Michelacci and Lopezsalido, 2007).

Based on the data from 2005 to 2013, our article examines the impact of Labor Contract Law on enterprise investment. The results of this article may be affected by the decline of corporate exports caused by the financial crisis in the United States. Compared with before the financial crisis, the export of Chinese enterprises declined remarkably after the financial crisis, which may lead to a decline in the level of enterprise investment, and then slow down China’s economic growth. In order to exclude this possible explanation, this article carries out the following tests: On the one hand, if the decline of enterprise export caused by the financial crisis plays a role in the reduction of enterprise investment, then this effect mainly occurs in the enterprises with export. On the other hand, if the decline in exports caused by the financial crisis leads to the decline in China’s regional economic growth, this effect will be stronger in regions with more exports. The results of this article exclude the impact of export decline caused by the US financial crisis on the research results.

This article has make following contributions: Firstly, it innovates the research on the economic consequences of LPL. Existing studies have analyzed the economic consequences of Labor Protection Law mainly from the viewpoint of enterprise labor cost, employment and cost stickiness (Banker et al., 2013). Taking the overseas market as a sample, a few literatures have studied the impact of employee dismissal costs on enterprise investment (Fairhurst and Serfling, 2015). With these foundation, this article analyzes the impact of China’s Labor Protection Law on enterprise investment at the micro level and regional economic growth at the macro level. Second, it provides a possible new explanation for China’s post-2008 decline in economic growth. As for the decline of China’s economic growth after 2008, existing studies have mainly explained it from the aspects of the American financial crisis and the weakening of demographic dividend. On the basis of these studies, this article provides a possible new explanation from the viewpoint of Labor Contract Law increasing the policy burden of private enterprises. Thirdly, it provides new evidence for state-owned enterprises to fulfill social goals (political goals). Lot of researches have shown that the implementation of social goals leads to the low efficiency of state-owned enterprises, but few literatures provide direct evidence for the social goals of state-owned enterprises. This article provides new evidence for state-owned enterprises to undertake more social goals, that is, although the Labor Contract Law will increase the burden of employees in private enterprises, this effect does not exist in state-owned enterprises with soft employee constraints. Finally, from the perspective of government macro policies, the relevant researches on enterprise micro behaviors are expanded (Julio and Yook, 2012).

The research in this article has important policy implications. A common problem faced by countries in transition is how to balance the relationship between economic growth and social harmony. This study shows that the implementation of the LPL, whose main goal is to promote social harmony, may increase the policy burden on the most dynamic private economy, reduce its employment flexibility and human resource allocation efficiency, inhibit its investment activities, and thus drag down China’s economic growth. Therefore, to maintain sustained and rapid economic growth, on the one hand, the government needs to formulate alternative policies, such as encouraging and supporting the innovation of enterprises, especially small and medium-sized enterprises, to hedge the increased policy burden; on the other hand, enterprises need to further strengthen technological innovation to alleviate the negative impact of social harmony goals on enterprises.

## Hypothesis Proposed

### Labor Contract Law, Property Right Nature, and Enterprise Investment

According to financial theory, program value is usually impact by the risk and the cash flow in the future (Miller, 1959). Under the same conditions, the smaller risk of program always means there will be more enterprise invests (Julio and Yook, 2012). On this basis, the scholars represented by Pindyck research shows that the management of the project management flexibility has an important value, namely, according to the project implementation, enterprise decision makers has the right to expand projects and reduce projects, when the project is in good condition, the decision makers expand the project, resulting in the value of project growth options, and the project reduce the value of project liquidation options (Abel, 1995; Pindyck, 1986). At the same time, enterprise decision makers have the right to reconfigure the resources related to the project. Therefore, the higher the flexibility of project management, the greater the investment value, and the more the enterprise investment.

Unlike the 1994s Labor Law, the Labor Protection Law enacted in 2008 has a major impact on enterprise labor costs and human resources allocation in the following aspects: Firstly, enterprises will face greater penalties for illegal labor costs, such as failure to sign formal labor contracts, failure to pay the full five insurances and housing fund for employees, and illegal dismissal of employees. Secondly, the provisions in the Labor Contract Law, such as open-ended labor contracts and economic compensation for the termination of labor contracts by consensus between employers and employees, will significantly reduce the flexibility of enterprises in employment, thus not conducive to the allocation efficiency of enterprises’ human resources (Fairhurst and Serfling, 2015). Thirdly, it may affect the working efficiency of employees. On the one hand, the Labor Contract Law increases employees’ sense of job security, which may promote more investment in enterprise-specific knowledge and thus increase employees’ productivity (Belot et al., 2007). Other way, the Labor Protection Law reduces the cost of punishment faced by employees, which may lead to the lethargy effect of protecting the slacker, ultimately detrimental to employees’ motivation and productivity (Belot et al., 2007; Rühmann and Südekum, 2010). So, the impact of Labor Protection Law on employees’ work efficiency is a double-edged sword, which may largely depend on enterprise culture.

Meanwhile, facing the impact of LPL, enterprises may take the initiative to respond: On the one hand, further strengthen the construction of corporate culture to encourage employees to invest more proprietary knowledge and improve their work efficiency, but these measures may require enterprises to have a good corporate culture foundation. On the other hand, to reduce the impact of Labor Protection Law on enterprise labor costs, enterprises can carry out factor substitution, which requires enterprises to have strong financial strength as the basis. We believe that there may be a period of adjustment for enterprises that have the conditions, that is, it will take some time to improve the enterprise culture and make breakthroughs in technological innovation in the equipment industry. As a result, the LPL may have a period of “pain” on such enterprises.

To sum up, the LPL will cause changes in four aspects: the cost of illegal labor; Reduce flexibility of employment; Employee efficiency; Replace human labor with more advanced equipment. These may have an impact on corporate investment. Drawing on the ideas and logic of Pindyck (1986), Abel (1995), we construct the model of the value of an investment project as follows:


(1) V=NPV+Option_growth+Option_switch+Option_put

The intuitive economic meaning is that the project value includes the following four aspects: the first is the net present value of the project, that is, the discounted present value of the future cash flow of the project under a given investment scale; The second is the value of project growth option, that is, enterprise decision makers have the expand right to improve the value of the project if the project is favorable in the future. The third is the value of project conversion option, that is, with the change of the project environment, enterprise decision makers have the right to reconfigure human resources and other resources to improve the value of the project, such as introducing appropriate human resources from outside to replace the existing human resources; The fourth is the value of project shrinkage or liquidation option, that is, in the case of poor future environment of the project, the enterprise decision maker has the right to reduce or liquidate the value brought to the enterprise by the project, which is usually accompanied by liquidation of fixed assets and dismissal of personnel.

According to model (1), we first analyze the impact of illegal labor costs, labor flexibility, and employee efficiency on project value and enterprise investment. First, under the condition of the illegal enterprise employee, the Labor Contract Law would increase the cash outflow of enterprises violation, at the same time, increase the risk of enterprise employee dismissal and costs, and reduce the project’s net present value, growth option value, the conversion option value and contract option value, could reduce the enterprise investment level. Secondly, the decline of the flexibility of the enterprise will hinder the enterprise to readjust human resources according to the environment of the project, and then reduce the value of the conversion option and contraction option of the project, and finally reduce the investment level of the enterprise (Rogerson, 1993; Fairhurst and Serfling, 2015). At the same time, when the profit expectation of the project is reduced, the difficulty and cost of firing the employees increase, which leads to the stickiness of the project employee cost (Banker et al., 2013), thus increasing the operation risk of the project and reducing the investment level of the enterprise. Finally, the improvement of employee efficiency will increase the net present value of the project and the value of the growth option of the project, thus increasing the investment level of the enterprise (Belot et al., 2007; Fairhurst and Serfling, 2015). The above analysis shows that the conditions for the improvement of employees’ working efficiency under the LPL are as follows: before the implementation of the LPL, the company has a good foundation of corporate culture, and the implementation of the LPL will further enhance the company’s corporate culture. Otherwise, the LPL could encourage the slacker effect. In China, the management of small and medium-sized private enterprises is usually not very formal and may lack a good foundation of corporate culture. Therefore, the Labor Contract Law will reduce the enthusiasm and efficiency of employees. For listed companies with relatively good corporate culture foundation, the improvement of staff efficiency by Labor Contract Law may be in a period of “labor pain,” because it takes some time to optimize corporate culture.

In the face of the impact of LPL, if an enterprise adopts factor substitution, its impact on enterprise investment is as follows: On the one hand, Labor Contract Law reduces the power of enterprise decision makers to allocate projects freely, especially, it significantly reduces the value of contract options of projects, thus leading to the decline of investment value of projects. Other way, when the production and sales scale of the project remains unchanged, the fixed asset investment of the enterprise will increase when the enterprise replaces labor with equipment that with better performance but higher price. Generally speaking, factor substitution may lead to the increase of enterprise investment, but the following conditions should be met: the enterprise has strong financial strength and the strength to purchase equipment with high performance and excellent price; Companies have not yet adopted good equipment to reduce human capital, and the price of the equipment is reasonable. Before the Labor Contract Law, under the influence of the reduction of China’s demographic dividend, the cost of human capital in China has shown a high growth trend. With sufficient financial strength, enterprises may have adopted equipment with good performance to reduce the use of human capital. In this case, Enterprises’ decision of factor substitution may be subject to the development and breakthrough of equipment industry technology. Therefore, we believe that: for private non-listed companies, due to financing constraints, they may not be able to invest more capital for factor replacement; For listed companies, before the LPL, they may have adopted equipment with good performance to alleviate the rapid growth of labor costs. At this time, it needs to wait for the technological breakthrough of the equipment industry to carry out factor substitution, that is, there is a “labor pain” period for listed companies to carry out factor substitution.

To sum up, the influence of the Labor Protection Law on enterprise investment is uncertain: According to the view of illegal costs and flexible employment, Labor Contract Law will reduce enterprise investment; According to the view of employee efficiency and factor substitution, Labor Contract Law may increase enterprise investment, but this effect has a “labor pain” period, and this effect is mainly concentrated in the listed companies with good corporate culture foundation and relatively strong financial strength.

Studies have shown that in the process of corporatization reform of state-owned enterprises in China, the government gradually decentralized its power, but still retained the power of acquisition and merger, appointment and dismissal of senior executives. Therefore, in order to obtain better career prospects, executives will actively or passively fulfill social goals (political goals), such as hiring more employees. Nevertheless, This article puts forward the H1 to H3:

Hypothesis 1: The LPL has no significant impact on the investment of state-owned enterprises;

Hypothesis 2: The influence of Labor Protection Law on private listed companies’ investment has a “labor pain” period, that is, the negative impact of Labor Protection Law on private listed companies’ investment only occurs in the early implementation stage;

Hypothesis 3: The Labor Contract Law will significantly reduce the investment level of private non-listed companies.

### Labor Contract Law, Property Right Nature and Enterprise Investment − the Impact of Different Types of Enterprises

This article further analyzes whether there are differences in the influence of Labor Protection Law on enterprise investment between listed and non-listed private enterprises in different industries with different labor density. Compared with non-labor-intensive industries, labor-intensive projects need more employees, and labor cost and human resource allocation efficiency have a greater impact on the net present value, growth option value, conversion option value and contraction option value of the project, so the LPL has a greater impact on enterprise investment. Specifically, for private non-listed companies, compared with non-labor-intensive industries, the LPL has a greater negative influence on the illegal costs and labor activity of labor-intensive enterprises, and thus has a greater negative impact on enterprise investment. For private listed companies, in the early implementation of the LPL, the LPL has a greater negative effect on the flexibility of employment of enterprises in labor-intensive industries. Meanwhile, the countermeasures of enterprises have not been formed, which will ultimately have a greater negative impact on enterprise investment. Furthermore, the cost of violating laws and regulations in employment of private non-listed companies is different in enterprises of different sizes, which is more prominent in small-scale private enterprises. At the same time, small-scale private enterprises have more flexible employment. In summary, this article proposed the Hypothesis 4: At the initial stage of implementation, the negative effect of LPL on the investment of private listed companies is stronger in labor-intensive industries;

Hypothesis 5: The negative effect of LPL on investment in private non-listed companies is stronger in labor-intensive industries and small-scale enterprises.

## Methodology

### Data

For listed companies, based on the samples from 2005 to 2013, this article conducts the following screening: sample companies in the financial and insurance industries are excluded; Sample companies whose asset-liability ratio exceeded 100% before the implementation of LPL were excluded; Sample companies whose enterprise nature could not be determined were eliminated; To reduce the impact of initial public offerings, all companies should be listed for at least 1 year. To test the influence of the LPL on enterprise investment, we need to compare the investment level of sample companies in several years before and after the LPL. Therefore, the sample companies were all listed companies from 2005 to 2013. At the same time, to test whether there is a significant difference in the influence of the Labor Protection Law on the investment of enterprises with different property rights, we exclude the samples with changes in the property rights of enterprises from 2005 to 2010. The data employed in this article mainly include enterprise investment data, enterprise nature data and enterprise characteristic data. These data are from the CSMAR database.

For unlisted companies, our article selected the data of Chinese industrial enterprises from 2005 to 2009 as the initial sample, and then screened them according to the following criteria: (1) Excluded sample companies with asset-liability ratio over 100% before the implementation of Labor Contract Law. (2) Exclude foreign-funded enterprises and sample companies whose enterprise nature cannot be determined. In this article, We use two methods to judge the property right nature of enterprises, one is based on the type of enterprise registration, the other is based on the capital structure of enterprises. (3) Sample companies with less than 5 years of establishment and less than RMB 5 million in annual sales are excluded. Since the statistical scope of China Industrial Enterprise database is the manufacturing enterprises with sales of more than 5 million yuan in Mainland China, if the sales of a company is less than 5 million yuan, it indicates that the company may have data statistical errors or the company takes the initiative to be counted, so we remove it. (4) Exclude sole proprietorship and partnership. For sole proprietorship and partnership, there is a large overlap of investors and employees, and the Labor Protection Law has a weak influence on them. (5) Before and after the implementation of the LPL, enterprises are counted in the Chinese industrial enterprises database.

### Method

For listed companies, we set the regression model to be tested as:


$$I\,n\,v\,e\,s\,t=\alpha +\beta_{1}\,L\,a\,b\,o\,r\,\_\,l\,a\,w+\beta_{2}\,L\,a\,b\,o\,r\,\_\,l\,a\,w*p\,r\,i\,v\,a\,t\,e$$

(2) $$+\beta_{3}\,P\,o\,s\,t\,\_\,l\,a\,w+\beta_{4}\,P\,o\,s\,t\,\_\,l\,a\,w*p\,r\,i\,v\,a\,t\,e+\beta_{5}\,X+\varepsilon_{i\,t}$$

Invest is the explained variable, representing the investment level of listed companies. According to existing studies (Foster and Rosenzweig, 1996), our article adopts the following four methods to measure the investment level of enterprises:


$$\left(1\right)\,\mathrm{Invest1}:\frac{\mathrm{Fixed}\,\mathrm{assets},\mathrm{intangible}\,\mathrm{assets}\,\mathrm{and}\,\mathrm{other}\,\mathrm{long}-\mathrm{term}\,\mathrm{assets}}{\mathrm{Total}\,\mathrm{assets}}$$

$$\left(2\right)\,\mathrm{Invest2}:\frac{\mathrm{Fixed}\,\mathrm{assets},\mathrm{intangible}\,\mathrm{assets}\,\mathrm{and}\,\mathrm{other}\,\mathrm{long}-\mathrm{term}\,\mathrm{assets}}{\mathrm{Total}\,\mathrm{assets}}$$

(3) *Invest3*, defined as (cash paid for the construction of fixed assets, intangible assets and other long-term assets - net cash recovered from the disposal of fixed assets, intangible assets and other long-term assets)/total assets at the beginning of the period; (4) *Invest4*, Defined as (cash paid for the construction of fixed assets, intangible assets and other long-term assets + net cash paid by subsidiaries and other business units - net cash recovered from the disposal of fixed assets, intangible assets and other long-term assets - net cash recovered from the disposal of subsidiary and other business units)/ total assets at the beginning of the period.

The *Invest1* and *Invest2* test results are reported in the body. while the *Invest3* and *Invest4* test results are explained in the robustness test.Labor_law is the dummy variable in the early implementation of LPL, and it is 1 from 2008 to 2010, otherwise it is 0. Post_law is the dummy variable of the late implementation of LPL, which is 1 from 2011 to 2013, or 0 otherwise. private is a dummy variable of the property right nature of an enterprise, and if the ultimate controller of the company is not a government agency at all levels, private is 1; or else, it is 0. X is a vector composed of multiple control indicators. According to relevant research on existing enterprise investment (Duchin et al., 2009; Julio and Yook, 2012), this article considers the following influencing factors: (1) Enterprise characteristic variables. Enterprise Size (Size), defined as the natural logarithm of a company’s total assets. Asset-to-liability ratio (Leverage), defined as the ratio of total liabilities to total assets. Growth (Q), defined as the ratio of the market value of the total assets to the book value. Company cash holding level (Cash), defined as the ratio of cash level to total assets. Cash flow from operating activities (OCF), defined as the ratio of cash flow generated from business activities to the total assets at the beginning of the period. Years of establishment (Lnage), defined as the natural logarithm of years of establishment. In addition to the years of establishment and cash flow generated by business activities, other company characteristic variables lag one period. (2) Corporate governance variables. Actual controller usufruct ratio (Cashflow_right), defined as the cash flow right ratio of the actual controller lagging one period. The degree of separation of the actual controller (Divergence) is defined as the difference among the control right ratio and the cash flow right ratio of the actual controller of the lag period. (3) Industry variables. According to the industry classification code of CSRC, in addition to the manufacturing industry is classified by the secondary code, other industries are classified by the primary code, and agriculture as the benchmark industry.

For unlisted companies, we set the regression model to be tested as:


(3) $$I\,n\,v\,e\,s\,t\,3=\alpha +\beta_{1}\,L\,a\,b\,o\,r_{l\,a\,w}+\beta_{2}\,L\,a\,b\,o\,r\,\_\,l\,a\,w*p\,r\,i\,v\,a\,t\,e+\beta_{3}\,X+\varepsilon_{i\,t}$$

Among them, Invest3 is the explained variable, representing the investment level of unlisted companies. Referring to the measurement index of the investment level of listed companies, we adopt the ratio of the added value of fixed assets to the total assets at the beginning of the period to measure it. X is a vector composed of multiple control variables. With reference to the control variables of investment of listed companies, the following influencing factors are considered in this article when data is available: enterprise size (Size), defined as the natural logarithm of total assets of a company. Leverage, defined as the ratio of total liabilities to total assets. Growth (Q) is defined as the growth rate of the enterprise’s main business income. Return on assets (ROA), defined as the ratio of net income to total assets. Company life (Lnage) is defined as the natural logarithm of company life. The dummy variable (Industry) is determined by the first two characters of the industry code.

To verify the hypothesis 4, listed companies are divide into companies in labor-intensive industries and companies in non-labor-intensive industries, and then makes regression on model (2), respectively. In order to avoid the influence of the Labor Contract Law on enterprise employment, this article uses the relevant indicators of various industries at the end of 2006 as the standard to classify labor-intensive industries (Duchin et al., 2009). Specifically, taking the median number of employees per unit asset (Labor1 = total number of employees/total assets) or employee salary per unit sales (Labor2 = employee salary/operating income) of enterprises in the industry as the standard, the dummy variable YG1 (YG2) of whether the industry is labor-intensive is constructed on this basis. First, the median of Labor1 (Labor2) in each industry is calculated. On this basis, the median of all industries is calculated. If the median of Labor1 (Labor2) in an industry is greater than the median of all industries, it is a labor-intensive industry, YG1(YG2) is 1, otherwise it is 0.

To test hypothesis 5, we divide the sample of industrial enterprises into companies in labor-intensive industries and those in non-labor-intensive industries, and then perform regression on model (3), respectively. We take the number of employees per unit asset or the number of employees per unit sales of an enterprise as the relevant index for the division of labor-intensive industries, and then construct the dummy variable YG5(YG6) for whether it is a labor-intensive industry in accordance with the similar method of listed companies. At the same time, according to the size of enterprises, we divide the sample of industrial enterprises into large companies and small companies, and then conduct regression test on model (3), respectively. To avoid the influence of the Labor Contract Law on enterprise scale, our article takes the total assets or net assets of enterprises at the end of 2006 as the standard for dividing enterprise scale. Specifically, SIZE1(SIZE2) is 1 if the company’s total assets (net assets) are greater than the median of all companies, and 0 otherwise.

The data set in our research are panel data with a small time span and a large number of cross-sectional observations. Therefore, in the regression of the model, we conduct clustering adjustment on the enterprise level for the standard error (Petersen, 2009). At the same time, To exclude the influence of outliers on test results, Winsorize all continuous variables at the highest and lowest 1% level.

### Descriptive Statistical Characteristics

Table 1 lists the descriptive statistical characteristics of the main variables. The statistics of the sample of listed companies show that the mean value and standard deviation of *Invest1* are 0.066 and 0.078, respectively, and the mean value and standard deviation of *Invest2* are 0.071 and 0.082, respectively, which indicates that there is a great difference in the investment level among Chinese listed companies. The mean, median and standard deviation of Labor_law were 0.334, 0.000 and 0.472, respectively, and the related indexes of Post_law were similar. The average value of private is 0.319, which indicates that nearly 1/3 of the sample companies in this article are private enterprises. The mean and standard deviation of the sample investment of industrial enterprises are 0.062 and 0.291, respectively, indicating that the investment level of non-listed companies is very different.

**TABLE 1:** Descriptive statistics of main variables.

	Number of samples	Minimum value	Median	Average value	Maximum value	Standard deviation
**Panel A: Sample of listed companies**						
Invest1	10452	0.000	0.041	0.066	0.446	0.078
Invest2	10452	0.000	0.044	0.071	0.470	0.082
Labor_law	10460	0.000	0.000	0.334	1.000	0.472
Private	10460	0.000	0.000	0.319	1.000	0.466
Labor_law*private	10460	0.000	0.000	0.106	1.000	0.308
Post_law	10460	0.000	0.000	0.333	1.000	0.471
Post_law*private	10460	0.000	0.000	0.106	1.000	0.308
**Panel B: Sample of industrial enterprises**						
Invest5	186189	–0.527	0.000	0.062	2.762	0.291
Labor_law	194472	0.000	0.000	0.384	1.000	0.486
Labor_law*private	194472	0.000	0.000	0.313	1.000	0.464

## Results and Analysis

### Labor Contract Law, Property Right Nature, and Enterprise Investment

Table 2 lists the test results of the Labor Contract Law, property right nature and enterprise investment. The test results of column (2) and Column (4) show that Labor_law and Post_law have no significant influence on the investment of listed companies. These show that compared with before the implementation of the LPL, the investment level of state-owned listed companies have not changed significantly in the early and late implementation of the LPL, which supports research hypothesis 1. The regression coefficients of Labor_law* private were −0.012 and −0.013, respectively, and both were significant at the statistical level of 1%. That indicates that in the early implementation of the Labor Contract Law, the negative impact of the Labor Contract Law on the investment of private listed companies is significantly higher than that of state-owned listed companies. By comparing the regression coefficient of Labor_law and Labor_law* private, as well as the F test of the sum of the two (test of B_1_ + B_2_ = 0), it can be found that in the initial implementation of Labor Contract Law, The regression results of Post_law and Post_law* private show that in the later period of the implementation of LPL, the LPL has no significant impact on the investment of private listed companies. These results verify hypothesis 2.

**TABLE 2:** Labor Contract Law (LCL), property right nature and enterprise investment.

Variable name	Sample of listed companies				Sample of industrial enterprises	
		
	Invest1		Invest2		Invest3	
			
	(1)	(2)	(3)	(4)	(5)	(6)
Labor_law (B_1_)	−0.003 (−0.932)	0.001 (0.386)	−0.003 (−0.921)	0.001 (0.424)	−0.032*** (−20.157)	−0.002 (−0.729)
Labor_law* private (B_2_)		−0.012*** (−3.087)		−0.013*** (−3.137)		−0.039*** (−12.648)
Post_law (B_3_)	0.002 (0.359)	0.002 (0.410)	−0.002 (−0.385)	−0.002 (−0.318)		
Post_law* private (B_4_)		−0.001 (−0.305)		−0.001 (−0.190)		
Size	−0.014*** (−5.681)	−0.014*** (−5.763)	−0.015*** (−5.360)	−0.015*** (−5.438)	0.271*** (53.010)	0.273*** (53.288)
Leverage	−0.044*** (−5.712)	−0.044*** (−5.735)	−0.049*** (−5.941)	−0.050*** (−5.929)	−0.162*** (−17.213)	−0.163*** (−17.411)
Q	0.008*** (6.386)	0.008*** (6.593)	0.009*** (6.497)	0.009*** (6.670)		
Growth					0.047*** (20.295)	0.047*** (20.217)
Cash	0.088*** (6.785)	0.087*** (6.745)	0.106*** (7.593)	0.104*** (7.544)		
OCF	0.070*** (3.676)	0.071*** (3.751)	0.081*** (3.900)	0.082*** (3.976)		
ROA					−0.228*** (−18.043)	−0.225*** (−17.825)
Cashflow_right	0.000 (0.475)	0.000 (0.658)	0.000 (0.664)	0.000 (0.839)		
Divergence	−0.000 (−0.378)	−0.000 (−0.295)	−0.000 (−0.252)	−0.000 (−0.161)		
Lnage	−0.017* (−1.668)	−0.016 (−1.633)	−0.005 (−0.495)	−0.005 (−0.463)	−0.113*** (−23.104)	−0.108*** (−22.221)
B_1_+B_2_ = 0		9.57***		9.38***		523.74***
B_3_+B_4_ = 0		0.01		0.20		
Observations	9830	9830	9830	9830	186098	186098
R^2^	0.073	0.075	0.072	0.074	0.121	0.122

Due to space constraints, results for the constant terms are not listed (same below). *** and * means of 1% and 10% significance levels, respectively, and the numbers in brackets are t-values.

The test results of column (5) show that the regression coefficient of Labor_law is −0.032, and it is fundamentally at the statistical level of 1%. This suggests that, overall, the LPL will significantly reduce the level of investment by industrial enterprises. By comparing the test results of listed companies, we find that the Labor Protection Law has a stronger impact on the investment of non-listed companies than listed companies. The possible reason lies in that illegal costs and labor flexibility have a greater impact on unlisted companies. The test results in Column (6) show that the Labor Protection Law has no fundamentally influence on the investment of state-owned non-listed companies. By comparing the regression coefficient of Labor_law and Labor_law* private and the F test of their sum (test of B_1_ + B_2_ = 0), we could found that the Labor Protection Law will fundamentally reduce the investment level of private non-listed companies, which supports hypothesis 3.

### The Labor Contract Law, Property Right Nature and Enterprise Investment − Based on the Grouping Test of Different Types of Enterprises

Here we further examine whether the impact of the LPL on enterprise investment is significantly different among different types of companies. Table 3 lists the test results of listed companies. The odd columns show the impact of the LPL on investment in non-labor-intensive industries. The outcomes show that in non-work escalated businesses, the Labor Protection Law essentially affects the venture of recorded organizations. The even columns show the impact of the LPL on business investment in labor-intensive industries. The results show that in labor-intensive industries, the LPL has no significant impact on the investment of state-owned listed companies, but it will significantly reduce the investment level of private listed companies. These experimental outcomes are steady with the assumptions for theory 4.

**TABLE 3:** The Labor Protection Law (LPL), property right nature and enterprise investment − based on the grouped test of listed company employees.

Variable name	Invest1				Invest2			
		
	(1)	(2)	(3)	(4)	(5)	(6)	(7)	(8)
	YG1 = 0	YG1 = 1	YG2 = 0	YG2 = 1	YG1 = 0	YG1 = 1	YG2 = 0	YG2 = 1
Labor_law (B_1_)	0.001 (0.204)	0.000 (0.109)	−0.002 (−0.546)	0.006 (1.111)	0.001 (0.176)	0.001 (0.235)	−0.003 (−0.592)	0.007 (1.171)
Labor_law* private (B_2_)	−0.007 (−1.283)	−0.015*** (−2.771)	−0.005 (−0.942)	−0.024*** (−3.871)	−0.008 (−1.269)	−0.016*** (−2.851)	−0.006 (−1.109)	−0.025*** (−3.636)
Post_law (B_3_)	−0.000 (−0.048)	0.002 (0.305)	−0.000 (−0.016)	0.004 (0.573)	−0.007 (−0.930)	0.001 (0.170)	−0.003 (−0.459)	−0.001 (−0.105)
Post_law* private (B_4_)	0.006 (0.840)	−0.006 (−0.959)	−0.000 (−0.019)	−0.003 (−0.357)	0.007 (0.886)	−0.006 (−0.848)	0.000 (0.079)	−0.002 (−0.256)
Size	−0.012*** (−3.129)	−0.018*** (−5.303)	−0.015*** (−5.250)	−0.014*** (−2.819)	−0.013*** (−3.140)	−0.018*** (−4.820)	−0.017*** (−5.459)	−0.011** (−2.154)
Leverage	−0.034*** (−2.920)	−0.055*** (−5.371)	−0.051*** (−5.045)	−0.035*** (−3.001)	−0.040*** (−2.945)	−0.060*** (−5.620)	−0.055*** (−4.854)	−0.042*** (−3.442)
Q	0.006*** (−2.859)	0.010*** (6.250)	0.009*** (5.614)	0.009*** (4.260)	0.007*** (3.148)	0.010*** (6.076)	0.010*** (5.881)	0.008*** (3.803)
Cash	0.087*** (5.143)	0.089*** (4.668)	0.094*** (5.614)	0.078*** (3.687)	0.097*** (5.302)	0.112*** (5.501)	0.108*** (6.123)	0.101*** (4.436)
OCF	0.051 (1.421)	0.075*** (3.478)	0.107*** (4.501)	0.018 (0.587)	0.065* (1.684)	0.112*** (3.596)	0.125*** (4.843)	0.020 (0.603)
B_1_+B_2_ = 0	1.68	8.46***	2.48	10.32***	1.66	7.99***	3.15*	8.03***
B_3_+B_4_ = 0	0.58	0.34	0.00	0.02	0.00	0.33	0.14	0.09
Observations	4293	5537	6068	3762	4293	5537	6068	3762
R^2^	0.059	0.091	0.088	0.063	0.060	0.089	0.092	0.058

Due to space limitations, the results of Cashflow_right, Divergence, Lnage, and constant terms are not listed. ***, **, and * represent significance levels of 1%, 5%, and 10%, respectively, and the numbers in brackets are t-values.

Table 4 lists the grouped test results for industrial enterprises. Columns (1) to (4) are the test results after grouping according to labor-intensive industries. The test results in columns (1) to (4) show that no matter in labor-intensive industries or non-labor-intensive industries, the LPL has no significant impact on the investment of state-owned non-listed companies. In the samples of different labor-intensive industries, the impact of LPL on the investment of private non-listed companies is significantly negative. Compared with non-labor-intensive industries, the LPL has a greater negative impact on the investment of private non-listed companies in labor-intensive industries. Columns (5) to (8) are the test results after grouping according to enterprise size. The test results in columns (5) to (8) show that the LPL has no significant impact on the investment of state-owned non-listed companies, no matter for large or small companies. In the samples of different sizes, LPL will reduce the investment level of private non-listed companies. By comparing the differences between groups, we find that the LPL has a greater negative impact on the investment of small-scale private non-listed companies than large companies.

**TABLE 4:** The LPL, property right nature and Enterprise investment − grouping test based on employee and size of industrial enterprise.

Variable name	Grouping by labor-intensive industries				Grouping by enterprise size			
		
	(1)	(2)	(3)	(4)	(5)	(6)	(7)	(8)
	YG1 = 0	YG1 = 1	YG2 = 0	YG2 = 1	YG1 = 0	YG1 = 1	YG2 = 0	YG2 = 1
Labor_law (B_1_)	−0.005 (−1.419)	0.003 (0.599)	−0.003 (−0.753)	−0.001 (−0.311)	0.001 (0.141)	−0.001 (−0.495)	0.003 (0.441)	−0.003 (−0.986)
Labor_law* private (B_2_)	−0.033*** (−8.064)	−0.046*** (−9.192)	−0.005 (−7.293)	−0.044*** (−10.287)	−0.051*** (−5.313)	−0.035*** (−10.697)	−0.050*** (−6.078)	−0.036*** (−10.691)
Size	0.275*** (33.843)	0.272*** (41.144)	0.268*** (33.264)	0.278*** (41.740)	0.344*** (41.256)	0.215*** (35.123)	0.311*** (38.751)	0.242*** (37.168)
Leverage	−0.147 *** (−10.007)	−0.175*** (−14.354)	−0.149*** (−9.998)	−0.174*** (−14.418)	−0.203*** (−13.69)	−0.133*** (−13.687)	−0.215*** (−14.54)	−0.118*** (−9.847)
Growth	0.046*** (13.535)	0.048*** (15.028)	0.050*** (14.098)	0.045*** (14.501)	0.036*** (9.860)	0.053*** (17.681)	0.031*** (9.033)	0.056*** (18.332)
ROA	−0.249*** (−12.323)	−0.210*** (−12.974)	−0.226*** (−12.13)	−0.224*** (−13.041)	−0.180*** (−10.71)	−0.264 *** (−13.977)	−0.182*** (−10.30)	−0.264*** (−14.829)
Lnage	−0.109*** (−14.175)	−0.108*** (−17.152)	−0.107*** (−13.60)	−0.110*** (−17.693)	−0.127*** (−12.99)	−0.098*** (−17.822)	−0.109*** (−13.17)	−0.107*** (−17.794)
B_1_+B_2_ = 0	178***	347***	205***	327***	230***	294***	242***	302***
Observations	80961	105137	77408	108690	70268	115830	73067	113031
R^2^	0.116	0.126	0.121	0.123	0.165	0.094	0.150	0.106

***, **, and * represent significance levels of 1%, 5% and 10%, respectively, and the numbers in brackets are t-values.

### The Labor Contract Law and Regional Economic Growth

This article further analyzes whether the negative impact of LPL on private enterprise investment will be transferred to regional economic growth. Table 5 lists the corresponding test results. Where, the explained variable is the GDP growth rate (%) of a certain region. Myjj1 (Myjj2) is a measure of the proportion of regional private economy, which is constructed according to the median of the proportion of fixed asset investment in non-state-owned economy in total fixed asset investment (the proportion of employment provided by non-state-owned economy in total urban employment) in the marketization index compiled by. In order to mitigate the impact of the LPL on regional indicators related to private economy, these indicators are based on the data of 2006. If the proportion of fixed asset investment in non-state-owned economy in total fixed asset investment (the proportion of employment provided by non-state-owned economy in total urban employment) in a certain region is above the median, Then Myjj1(Myjj2) is 1, otherwise 0.

**TABLE 5:** Labor Protection Law, proportion of private economy and regional economic growth.

Variable name	(1)	(2)	(3)	(4)	(5)	(6)
	All the samples	All the samples	*M*_*yjj*_1 = 0	*M*_*yjj*_1 = 1	*M*_*yjj*_2 = 0	*M*_*yjj*_2 = 1
Labor_law	−1.033* (−3.105)	−1.949* (−2.550)	−0.021 (−0.018)	−3.169* (−4.894)	−0.470 (−0.485)	−3.675* (−5.119)
Post_law	−2.448* (−5.773)	−4.692* (−3.470)	−2.360 (−1.006)	−6.353* (−8.609)	−3.194 (−1.362)	−6.777* (−6.927)
Lnhuman		6.849 (0.790)	12.726 (0.823)	−5.846 (−0.700)	13.067 (0.992)	−10.547 (−1.472)
Lncapital		1.080 (0.549)	−2.526 (−0.660)	3.915* (3.423)	−1.548 (−0.563)	5.494* (4.902)
Lninfra		0.941 (0.965)	1.839 (1.073)	0.106 (0.128)	1.357 (0.983)	0.839 (0.710)
Lnres		0.900* (2.779)	−0.096 (−0.125)	1.062* (4.007)	0.191 (0.379)	1.206* (3.530)
Market		−0.316 (−1.216)	−0.909* (−2.143)	−0.145 (−0.627)	−0.964* (−2.147)	0.002 (0.006)
Paygrowth		0.070 (0.018)	−5.561 (−0.966)	4.989 (1.107)	−5.476 (−1.040)	8.739* (2.150)
Observations	279	261	126	135	135	126
R2	0.277	0.341	0.180	0.645	0.256	0.615

* represent significance levels of 10%.

According to the existing literatures (Prest, 1959; Nazrul, 1998), we control other important factors affecting regional economic growth: Human capital (Lnhuman) is measured by the natural logarithm of education years per capita in provinces, autonomous regions or municipalities. Illiterate or semi-illiterate, primary school, junior middle school, senior high school, junior college and above education years are given 2, 6, 9, 12, and 16 years, respectively. Physical capital (Lncapital) is measured by the natural logarithm of per capita capital stock of a province, autonomous region or municipality directly under the Central Government, and capital stock is estimated by the “perpetual inventory method.” Transportation infrastructure (Lninfra) is measured by the natural logarithm of the sum of highway, railway and inland waterway mileage in provinces, autonomous regions or municipalities directly under the central government; Natural resources (Lnres) : adopt natural logarithmic measurement of energy output of provinces, autonomous regions or municipalities directly under the central government; The degree of marketization (Market) is measured by the score of “marketization index” in China’s regional marketization index system. Paygrowth is measured by the growth rate of average wages in provinces, autonomous regions or municipalities directly under the central government, so as to control the impact of labor supply and demand relationship or demographic dividend on regional economic growth.

The test results of columns (1) and (2) show that the regression coefficients of Labor_law and Post_law were negative and significant at the statistical level of 1% or 5%. These show that in the early and late implementation of the LPL, China’s regional economic growth declined significantly. Columns (3) to (6) test whether there are differences in the impact of LPL on regional economic growth in regions with different proportions of non-state-owned economic investment or employment. The test results in columns (3) and (4) show that the LPL has no significant impact on regional economic growth in regions with low proportion of fixed assets investment in non-state-owned economies. In the regions with high proportion of fixed assets investment in non-state-owned economy, the regional economic growth declined significantly in the early and late period of the implementation of the LPL. These test results show that the negative impact of the LPL on regional economic growth is mainly caused by reducing investment by private enterprises. The test results in Column (5) and Column (6) show that the LPL has no significant impact on regional economic growth in the regions with a low proportion of employment provided by non-state-owned economy. In regions with a high share of employment in the non-state sector, the LPL can significantly reduce regional economic growth. These test results show that the LPL will significantly reduce the investment level of private enterprises in labor-intensive industries, and then reduce the regional economic growth rate in the current period. Therefore, the negative impact of the LPL on private enterprise investment will eventually drag down China’s economic growth.

### Robustness Test

(1) Impact of the financial crisis. The research results of this article may be affected by the financial crisis in the United States. Since 2008, China’s export has experienced a significant decline. Therefore, if enterprises expect a significant decline in export in the future, they will slow down the expansion rate, further reduce the investment level, and China’s regional economic growth rate will also decline significantly. In order to exclude such a possible explanation, this article conducts the following robustness tests: On the one hand, if the decline of enterprise export caused by the financial crisis plays a role in the reduction of enterprise investment, then this effect mainly occurs in the enterprises with export. This article divides enterprises into export-oriented enterprises (Export = 1) and non-export-oriented enterprises (Export = 0) based on whether enterprises had overseas market sales before the financial crisis. On this basis, the samples with and without exits were tested again on Model (2), and the test results were shown in Table 6. The results show that the effect of the LPL on the investment of state-owned enterprises or private enterprises is not affected by whether the enterprises export goods, which to some extent excludes the impact of export decline caused by the US financial crisis on the research results of this article.

**TABLE 6:** Robustness test result.

Variable name	Invest1		Invest2	
		
	(1)	(2)	(3)	(4)
	Export = 0	Export = 1	Export = 0	Export = 1
Labor_law	0.003 (0.651)	−0.003 (−0.532)	0.001 (0.262)	0.001 (0.242)
Labor_law* private	−0.012** (−2.337)	−0.012* (−1.809)	−0.012** (−2.301)	−0.013** (−1.932)
Post_law	0.002 (0.315)	0.000 (0.030)	−0.005 (−0.800)	0.004 (0.492)
Post_law* private	0.004 (0.622)	−0.008 (−1.046)	0.004 (0.695)	−0.008 (−0.916)
Observations	6117	3713	6117	3713
R^2^	0.069	0.095	0.070	0.090

To save space, the results of other control variables and constant terms are not shown. ** and * represent significance levels of 5% and 10%, respectively, and the numbers in brackets are t-values for the two-tailed test.

On the other hand, if the decline in exports caused by the financial crisis leads to the decline in China’s regional economic growth, this effect will be stronger in regions with more exports. However, the results of this article show that the effect of the LPL on regional GDP growth rate is not affected by the proportion of regional exports. This further excludes the impact of export decline caused by the US financial crisis on the research results of this article.

(2) Monetary policy, fiscal policy and time trend effect. Since variables in the LPL were divided in 2008, there may be two alternative explanations for the test results in this article: On the one hand, the investment level of private enterprises may show a significant decline before the implementation of LPL, while state-owned enterprises do not show such a trend. Therefore, the test results of this article are only the result of time trend, rather than the effect of LPL. On the other hand, the impact of monetary and fiscal policy changes is typically 4 trillion yuan of investment implemented from the beginning of 2009 to the end of 2010. Therefore, the findings of this article may be caused by 4 trillion yuan of investment. To eliminate these alternative explanations, we analyze the effect of time trend effect on firm investment. The results show that before the implementation of the LPL, the investment expenditure of private enterprises does not show a downward trend, and the decline of investment expenditure only appears in the early implementation of LPL, which excludes the influence of time trend effect. At the same time, according to the impact logic of 4 trillion yuan investment, the investment level of both private and state-owned enterprises should increase significantly in 2009 and 2010, which is inconsistent with the test results, and thus excludes the effects of monetary and fiscal policies.

(3) Other robustness tests. First, the impact of income tax policy changes. By adding the actual income tax rate into the regression model and directly testing whether the impact of the LPL on enterprise investment is affected by the change of enterprise income tax rate, the results show that the impact of LPL on enterprise investment is not affected by the change of enterprise income tax rate. Second, the impact of labor supply and demand changes. By adding the measurement index of the change of labor supply and demand into the regression model, and directly testing whether the influence of the LPL on enterprise investment is affected by the change of labor supply and demand, we find that the influence of the LPL on enterprise investment is not affected by the change of labor supply and demand. Thirdly, control the investment level of one and two periods lag, adopt the dynamic panel data regression model, and the research results of this article remain unchanged. Fourth, using *Invest3* and *Invest4* as explained variables, the tests of the related tables are replicated and the test results remain unchanged. Fifthly, the unit asset wage (employee wage/total assets) or unit cost employee wage (employee wage/operating cost) is used to construct the dummy variable of whether the enterprise is a labor-intensive industry, and the test results remain unchanged.

## Conclusion

This article study the influence of LPL on enterprise investment and regional economic growth. Our results indicate that the Labor Protection Law has no significant influence on the investment of state-owned enterprises. In the early stage of the implementation of LPL, the Labor protection Law will significantly reduce the investment level of private enterprises, and this effect is more obvious in labor-intensive industries and small and medium-sized enterprises. However, in the later stage of the implementation of LPL, the impact of LPL on the investment of private listed companies is weak. These results show that the LPL increases the illegal costs of private enterprises, reduces the flexibility of employment, and ultimately reduces the investment level of enterprises.

In the face of the influence of the Labor Protection Law, the countermeasures of private listed companies have a hedging effect in the late implementation of LPL, that is, to improve the work efficiency of employees through the improvement of corporate culture, to reduce the use of human capital by substituting factors; However, these effects do not exist in state-owned enterprises with soft employee constraints. Finally, this article examines the influence of the LPL on regional economic growth, and finds that the LPL will significantly reduce the regional GDP growth rate in China, and this effect is mainly reflected in the regions where private enterprises provide more jobs and the proportion of private economy is high. This article takes China’s emerging markets as the background, on the one hand, expands the relevant research on labor market friction from the perspective of enterprise investment and economic growth, on the other hand, provides new evidence for state-owned enterprises to fulfill social goals.

Under the condition of China’s transition economy, the government and the market are simultaneously distributing economic resources. The government usually formulates relevant systems and regulations according to the goal of social harmony, which may increase the policy burden on enterprises, reduce the investment level of enterprises, and ultimately hinder the sustained and rapid growth of China’s economy. In the future, it is worth further studying what specific measures can be taken by enterprises to relieve these policy burdens, and whether these measures will have a significant impact on regional economic growth.

## Data Availability Statement

The datasets presented in this article can be obtained by contacting the corresponding author where appropriate. Requests to access the datasets should be directed to YL, liaoyina10@cdut.edu.cn.

## Author Contributions

The author confirms being the sole contributor of this work and has approved it for publication.

## Conflict of Interest

The author declares that the research was conducted in the absence of any commercial or financial relationships that could be construed as a potential conflict of interest.

## Publisher’s Note

All claims expressed in this article are solely those of the authors and do not necessarily represent those of their affiliated organizations, or those of the publisher, the editors and the reviewers. Any product that may be evaluated in this article, or claim that may be made by its manufacturer, is not guaranteed or endorsed by the publisher.
